# Physical understanding of the tropical cyclone wind-pressure relationship

**DOI:** 10.1038/s41467-017-01546-9

**Published:** 2017-11-08

**Authors:** Daniel R. Chavas, Kevin A. Reed, John A. Knaff

**Affiliations:** 1 Purdue University, Department of Earth, Atmospheric, and Planetary Sciences, 550 Stadium Mall Drive HAMP 3221, West Lafayette, IN 47907 USA; 2 School of Marine and Atmospheric Sciences, Stony Brook University, Stony Brook, NY 11794 USA; 3 NESDIS/STAR, CIRA/Colorado State University, Campus Delivery 1375, Fort Collins, CO 80523-1375 USA

## Abstract

The relationship between the two common measures of tropical cyclone intensity, the central pressure deficit and the peak near-surface wind speed, is a long-standing problem in tropical meteorology that has been approximated empirically yet lacks physical understanding. Here we provide theoretical grounding for this relationship. We first demonstrate that the central pressure deficit is highly predictable from the low-level wind field via gradient wind balance. We then show that this relationship reduces to a dependence on two velocity scales: the maximum azimuthal-mean azimuthal wind speed and half the product of the Coriolis parameter and outer storm size. This simple theory is found to hold across a hierarchy of models spanning reduced-complexity and Earth-like global simulations and observations. Thus, the central pressure deficit is an intensity measure that combines maximum wind speed, storm size, and background rotation rate. This work has significant implications for both fundamental understanding and risk analysis, including why the central pressure better explains historical economic damages than does maximum wind speed.

## Abstract (web summary)

Tropical cyclone intensity is commonly measured by both central pressure and maximum wind speed, yet the physical relationship between the two is not understood. Here the authors show that the central pressure is an intensity measure that depends on maximum wind speed and the product of storm size and background rotation rate.

## Introduction

The relationship between the central pressure deficit and peak near-surface wind speed in a tropical cyclone is a long-standing unsolved problem in tropical meteorology, one that has significant implications for both our physical understanding of the tropical cyclone as well as the communication and interpretation of hazard information for evaluating risk of damage and loss of life. Historically, both metrics have been employed as essentially interchangeable measures of tropical cyclone intensity (the Saffir-Simpson Hurricane Scale was modified to focus solely on peak wind speed in 2009^1^). Various empirical estimates of the relationship between the two quantities, termed the wind-pressure relationship (WPR), are commonly employed^2–7^. However, the lack of a physical understanding of the relationship between the two quantities is problematic in both operations and research. In operations, their interchangeable usage leads to confusion when communicating potential short-term risk to the public given that the potential for significant impacts depends on many factors beyond simply peak wind speed^8,9^. This issue is especially important for rare cases, such as Hurricane Sandy (2012), that exhibit significantly lower central pressures than is expected for the given peak wind speed^10^. In research, authors typically select one of the two metrics arbitrarily for analysis^11–13^, thereby rendering intercomparison of results across studies difficult. Moreover, in climate modeling studies, the wind-pressure scattergram is commonly used to compare the statistics of a simulated tropical cyclone climatology against the observational record as a validation test^14,15^ despite the absence of a physical foundation for interpreting the result of this comparison. Curiously, general circulation models at resolutions of 50–100 km are capable of reproducing the range of central pressure deficit values found in observations despite their inability to reproduce the upper end of the range of peak wind speeds^14,16,17^, perhaps due to variations in other relevant storm properties, such as storm size. Finally, in the context of risk, the use of dual metrics muddies the interpretation of historical trends of storm intensity, particularly at landfall^18–21^, as well as the statistical assessment of long-term risk that necessarily employ intensity-dependent damage functions^22–25^. Importantly, though, econometric analysis has found that the minimum central pressure is a better predictor of historical hurricane economic damages in the United States than maximum wind speed^26^, a finding that currently lacks a physical explanation.

Although physical understanding is currently lacking, the prevailing empirical model^6,7^ for this relationship trained on historical observations determined that the central pressure deficit depends principally on storm peak wind speed and secondarily on latitude and a normalized measure of storm size. The choice of their parameters were broadly motivated by gradient wind balance, which directly relates the low-level radial distributions of pressure and wind. This balance is central to extant tropical cyclone theory^27^ and has been shown to hold reasonably well for tropical cyclones in high-resolution global climate model simulations^28^ and in observations^29^. However, the prediction of the central pressure deficit by gradient wind balance has yet to be directly tested, nor has its implicit dependence on the nature of the low-level wind field been rigorously analyzed in pursuit of a simpler physical understanding that is both operationally accessible and consistent with prevailing empirical models.

Here we seek to understand the fundamental physics governing the central pressure deficit and its relationship to the low-level wind field. Beginning from gradient wind balance, we exploit recent advances in our understanding of the wind field to derive a simple theoretical prediction for this relationship that depends on maximum wind speed and a parameter that combines outer storm size and background rotation rate. We then test this theory across a hierarchy of models^30^ that spans a reduced-complexity global model simulation experiment, an Earth-like simulation, and historical observations and discuss the experimental utility of each rung for both testing theory and bridging the gap between idealized experiments and the real Earth. We find that the simple theory holds well across the hierarchy, indicating that this theory captures the fundamental dependence of the central pressure deficit for real storms in nature. This understanding of the relationship between central pressure and maximum wind speed can be used to improve the interpretation of real-time tropical cyclone observations of storm intensity and size as well as to better understand variability in tropical cyclones and long-term hazard risk in both the historical record and in model simulations of present and future climate states.

## Results

### Theory

The relationship between the radial distributions of pressure and azimuthal wind may be approximated from the radial momentum equation by assuming cylindrical gradient wind balance (GWB), i.e.

1 $$ - \frac{1}{\rho }\frac{{\partial P}}{{\partial r}} + \frac{{{v^2}}}{r} + fv = 0,$$

where *P* is air pressure, *r* is radius from the storm center, *v* is the azimuthal wind, *ρ* is air density, and *f* is the Coriolis parameter evaluated at the latitude of the storm center. Radial integration of this balance equation over the entire storm circulation yields a prediction for the central pressure deficit in a tropical cyclone,

2 $${\rm{\Delta }}P = {P_0} - {P_{\rm{m}}}$$

where *P*_m_ is the minimum central pressure near the surface and *P*_0_ is the environmental pressure at the outer edge of the storm^6^. Here we pursue this integration formally in combination with recent theoretical advances in our understanding of storm structure.

Equation (1) may be rephrased in terms of absolute angular momentum, $M = rv + \frac{1}{2}f{r^2}_{}^{}$, as:

3 $$ - \frac{1}{\rho }\frac{{\partial P}}{{\partial r}} + \frac{{{M^2}}}{{{r^3}}} - \frac{1}{4}{f^2}r = 0.$$

We non-dimensionalize Eq. (3) with

4 $$\tilde M = \frac{M}{{{M_0}}},$$

5 $$\tilde r = \frac{r}{{{r_0}}},$$

6 $$\tilde P = \frac{P}{{{P_0}}},$$

7 $$\tilde \rho = \frac{\rho }{{{\rho _0}}},$$

where *r*_0_ is the outer radius of vanishing wind, ${M_0} = \frac{1}{2}fr_0^2$ is the angular momentum at *r* = *r*_0_, *P*_0_ is the air pressure at *r* = *r*_0_, and *ρ*_0_ is the air density at *r* = *r*_0_; the latter two may be considered the pressure and density of the ambient environment in which the storm is embedded. The result, following substitution of ${M_0} = \frac{1}{2}fr_0^2$, is

8 $$ - \frac{{4{P_0}}}{{{\rho _0}{f^2}r_0^2}}\frac{1}{{\tilde \rho }}\frac{{\partial \tilde P}}{{\partial \tilde r}} + \frac{{{{\tilde M}^2}}}{{{{\tilde r}^3}}} - \tilde r = 0.$$

From the Ideal Gas Law,

9 $$P = \rho {R_{\rm{d}}}{T_\rho },$$

where *R*_d_ is the dry gas constant and *T*_*ρ*_ is the density temperature, we may substitute $\frac{{{P_0}}}{{{\rho _0}}} = {R_d}{T_{\rho 0}}$. The final result is the following non-dimensional equation

10 $$ - \frac{1}{\beta }\frac{1}{{\tilde \rho }}\frac{{\partial \tilde P}}{{\partial \tilde r}} + \frac{{{{\tilde M}^2}}}{{{{\tilde r}^3}}} - \tilde r = 0,$$

with a single non-dimensional parameter, *β*, given by

11 $$\beta = \frac{{{{\left( {{\textstyle{1 \over 2}}f{r_0}} \right)}^{2}}}}{{{R_{\rm{d}}}{T_{\rho 0}}}}$$

Here we absorb the factor ${\textstyle{1 \over 4}}$ into the velocity scale $\frac{1}{2}f{r_0}$ in the numerator. This quantity is fundamental, as it represents the planetary tangential velocity intrinsic to the planetary angular momentum available to the tropical cyclone at the outer radius, i.e., ${M_0} = ({\rm{\Omega }}{\kern 1pt} {\rm{sin}}{\kern 1pt} (\phi ){r_0})\times{r_0}$, where Ωsin(*ϕ*) is the projection of the planetary rotation rate, Ω, onto the local vertical at latitude *ϕ*, and the second *r*_0_ is the moment arm of the storm.

Next, we seek analytical insight into the non-dimensional central pressure deficit, ${\rm{\Delta }}\tilde P = 1 - \frac{{{P_m}}}{{{P_0}}}$, where ${\rm{\Delta }}\tilde P$ is defined as a positive value following standard convention. Rearranging Eq. (10) and integrating from the storm center ($\tilde r = 0$) to the non-dimensional outer radius ($\tilde r = 1$) yields

12 $${\rm{\Delta }}\tilde P = \beta {\kern 1pt} {\int}_{0}^1 {\kern 1pt} \tilde \rho {\kern 1pt} \left( {\frac{{{{\tilde M}^2}}}{{{{\tilde r}^3}}} - \tilde r} \right){\kern 1pt} {\rm{d}}\tilde r,$$

where *β* is independent of $\tilde r$ and so may be pulled out of the integral. Thus, Eq. (12) dictates that $\Delta \tilde P$ is purely a function of $\tilde M(\tilde r)$, *β*, and radial variations in air density ($\tilde \rho$).

Recent work^31,32^ developed a solution for the complete $\tilde M(\tilde r)$ in a tropical cyclone that numerically merges analytical solutions for the convecting inner region^33^ and the non-convecting outer region^34^. Though this model does not have a closed-form analytical solution, the solution was shown to depend exclusively on three parameters: the maximum azimuthal-mean azimuthal wind speed, *V*_m_; $\frac{{{C_{\rm{d}}}f{r_0}}}{{{w_{{\rm{cool}}}}}}$, where *w*_cool_ is the radiative-subsidence rate in the non-convecting outer region and *C*_d_ is the surface momentum exchange coefficient; and the ratio of surface exchange coefficients of enthalpy and momentum in the inner region, $\frac{{{C_{\rm{k}}}}}{{{C_{\rm{d}}}}}$. These parameters were separated into two storm-specific parameters, *V*_m_ and *fr*_0_, which vary significantly in space and time both within the storm life-cycle and across storms, and two environmental parameters, $\frac{{{w_{{\rm{cool}}}}}}{{{C_{\rm{d}}}}}$ and $\frac{{{C_{\rm{k}}}}}{{{C_{\rm{d}}}}}$, which vary less strongly and in principle may be estimated in the absence of an actual storm (though whose values could be modified by the storm itself). Thus, the non-dimensional radial structure of $\tilde M$ is principally controlled by two velocity scales: *V*_m_ and *fr*_0_.

We now extend this analysis to our solution for $\Delta \tilde P$ given by Eq. (12). We first separate *β* itself into the product of the same storm parameter identified from the wind structure model (now including the factor $\frac{1}{2}$), $\frac{1}{2}f{r_0}$, and the environmental parameter *R*_d_ *T*_*ρ*0_. Thus, Eq. (12) dictates that $\Delta \tilde P$ depends principally on *V*_m_, $\frac{1}{2}f{r_0}$, and $\tilde \rho$; it depends secondarily on the environmental parameters $\frac{{{w_{{\rm{cool}}}}}}{{{C_{\rm{d}}}}}$, $\frac{{{C_{\rm{k}}}}}{{{C_{\rm{d}}}}}$, and *R*_d_ *T*_*ρ*0_. Variations in $\tilde \rho$, which represents density variations relative to the ambient environment, are small relative to the much larger variations in intensity (*V*_m_), latitude (*f*), and storm size (*r*_0_) exhibited by tropical cyclones on Earth, and thus $\tilde \rho$ may be taken as constant (shown below). Finally, translation of the non-dimensional central pressure deficit, $\Delta \tilde P$, to the traditional dimensional central pressure deficit, Δ*P* = *P*_0_ − *P*_m_ depends only on the environmental pressure *P*_0_. Because Δ*P* is typically at least an order of magnitude smaller than *P*_0_ (10–100 hPa and 1000 hPa, respectively), variations in $\Delta \tilde P$ and Δ*P* are approximately equivalent. A credible estimate of *P*_0_ is important specifically for the precise estimation of *P*_m_, though space-time variation of *P*_0_ is relatively small compared to that of *P*_m_. Similarly, space-time variation in *T*_*ρ*0_ may also be assumed to be relatively small, though its variation due to changes in sea surface temperature (e.g., in space or under climate change) could in principle be accounted for externally assuming constant boundary layer relative humidity within the tropics.

Thus, theoretically the central pressure deficit should depend principally on the velocity scales *V*_m_ and $\frac{1}{2}f{r_0}$, i.e.,

13 $${\rm{\Delta }}P \approx {\cal F}\left( {{V_{\rm{m}}},\frac{1}{2}f{r_0}} \right).$$

Specifically, the central pressure deficit increases with increasing intensity, size, and Coriolis parameter, though the precise quantitative dependence lacks an analytical solution and so is the subject of the remainder of this manuscript. This result is consistent with the prevailing empirical model^6^, which takes intensity, size, and latitude as predictors for the central pressure deficit. This also provides a physical basis for understanding why particularly large storms in nature have been observed to possess abnormally large central pressure deficits despite modest peak wind speeds.

### Hierarchy of models

**Fig. 1:**
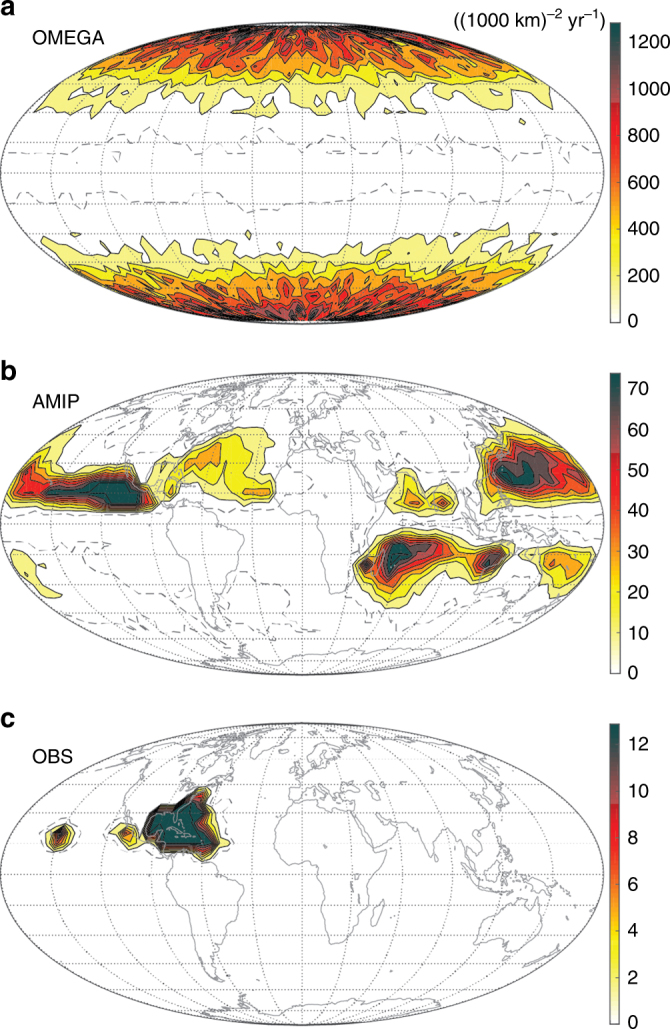
Storm count density across simulations and observations. **a** OMEGA. **b** AMIP. **c** Observations. Density is defined as number of track points per (1000 km)^2^ per year, calculated from data binned into lat-lon boxes of side length 5° weighted by the cosine of the box central latitude. Maximum value set to 99th percentile for clarity; gray dashed line denotes zero density contour. No data filters are applied

**Fig. 2:**
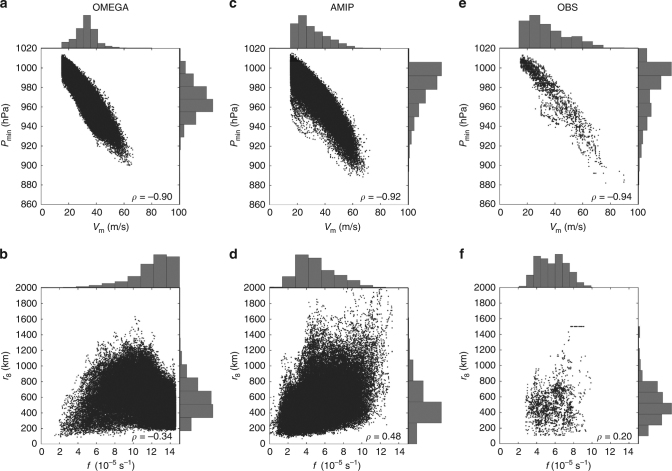
Joint distributions of key storm quantities across simulations and observations. **a** *P*_m_ and *V*_m_ in OMEGA. **b** *r*_8_ and *f* in OMEGA. **c**, **d** Same as **a**, **b** for AMIP. **e**, **f** Same as **a**, **b** for observations. Data set is filtered as described in the text

**Fig. 3:**
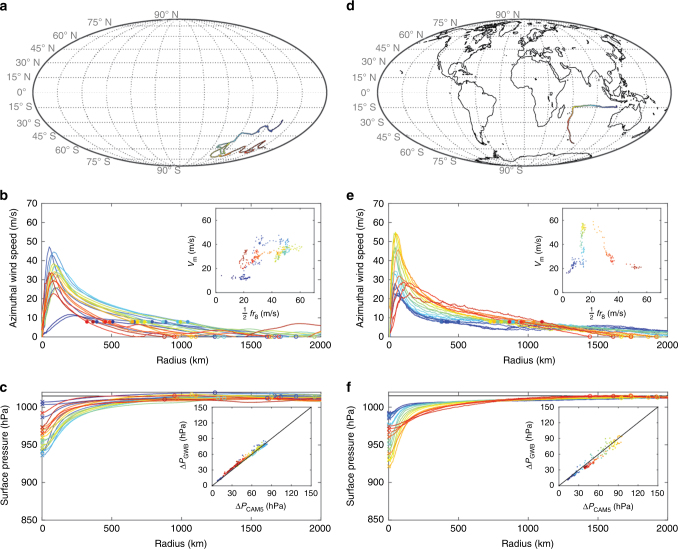
Example tracks and gradient wind balance calculations from OMEGA and AMIP simulations. **a** Track map for OMEGA. **b** Evolution of azimuthal-mean near-surface azimuthal wind profile and (inset) value of (*V*_m_, $\frac{1}{2}f{r_8}$) for OMEGA. **c** Azimuthal-mean surface pressure profiles and (inset) comparison of CAM5 central pressure deficit, Δ*P*_CAM5_, against gradient wind balance prediction, Δ*P*_GWB_ for OMEGA. **d**–**f** Same as **a**–**c** for AMIP track. Time increases from blue to green to red. Twenty profiles are plotted in even steps spanning the life-cycle; insets show all time steps. Filled circles denote *r*_8_; open circles denote *r*_0_ and *P*_0_; crosses denotes minimum central pressure

As described in Methods section, we test this theoretical prediction for Δ*P* across a hierarchy of models that spans a reduced-complexity aquaplanet simulation experiment (OMEGA), an Earth-like simulation (AMIP), and observations. The spatial distribution of storms and joint distributions of key quantities for both simulations and observations are provided in Figs 1, 2, respectively. We first test the prediction of the central prssure deficit directly from gradient wind balance. Example tracks and gradient wind balance calculations for OMEGA and AMIP are provided in Fig. 3. We then test how well this prediction can be replicated by a hierarchy of three simple multiple-linear regression (MLR) models that take as predictors: *V*_m_ only (standard wind-only baseline); *V*_m_, *f*, and the radius of 8 ms^−1^, *r*_8_ (analogous to the prevailing empirical model^6^); and *V*_m_ and $\frac{1}{2}f{r_8}$ (new theory).

**Fig. 4:**
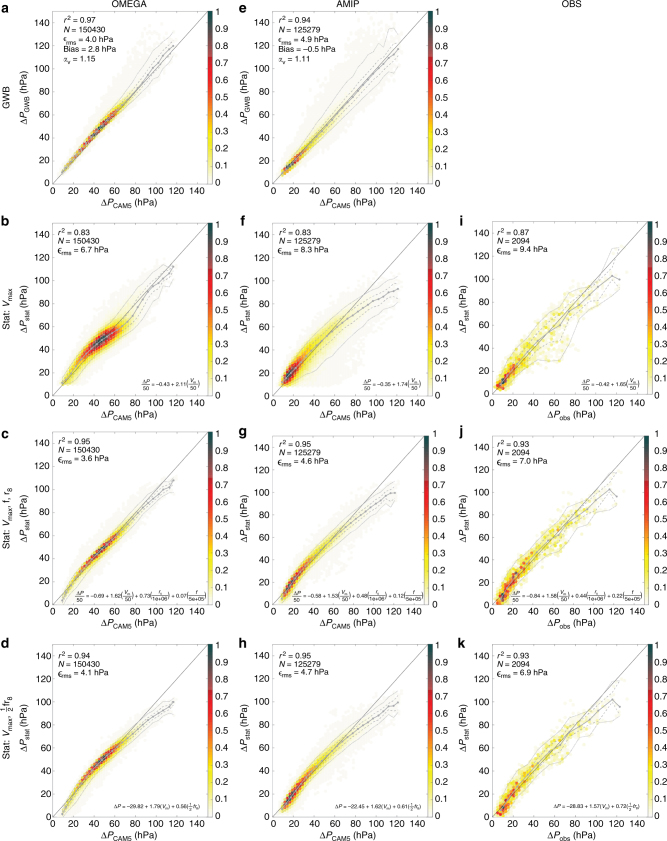
Predicted vs. actual Δ*P* (hPa) across simulations and observations. **a** Direct gradient wind balance model for OMEGA. **b** MLR model with predictor *V*_m_ for OMEGA **c** MLR model with three predictors (*V*_m_, *f*, *r*_8_) for OMEGA. **d** MLR model with two predictors (*V*_m_, $\frac{1}{2}f{r_8}$) for OMEGA. **e**–**h**, Same as **a**–**d** for AMIP. **i**–**k** Same as **b**−**d** for observations. Color denotes relative frequency within hexagonal bin. Black line denotes one-to-one correspondence. Gray lines denote median (solid), interquartile range (dashed), and 5–95% range (dotted) of predicted Δ*P* within 5 hPa bins of Δ*P*_CAM5_ and Δ*P*_obs_ starting from zero, plotted at bin-median value of each (dot) for bins with at least five data points. *N* denotes sample size. For GWB, mean prediction bias defined as ${\overline {\Delta P} _{{\rm{GWB}}}} - {\overline {\Delta P} _{{\rm{CAM5}}}}$, and *α*_*V*_ denotes wind speed rescaling factor

Figure 4 compares the model predictions of Δ*P* against their true values, Δ*P*_*CAM*5_, across simulations and observations for the direct gradient wind balance calculation as well as each of three MLR statistical models. The gradient wind balance calculation is not performed for observations as it requires a credible estimation of the entire wind profile out to large radii, a suitable database of which is not available. For the gradient wind calculations, the azimuthal wind profile has been multiplied by the constant factors (*α*_*V*_) of 1.15 and 1.11 for OMEGA and AMIP, respectively. These values yield a slope of approximately one for the linear fit between Δ*P*_GWB_ and Δ*P*_CAM5_. The square of the Pearson correlation coefficient, *r* ^2^, and the root-mean-square error, *ε*_rms_, are calculated as measures of performance for each model.

We begin with the simulations. The prediction Δ*P*_GWB_ performs very well in explaining the vast majority of variance in Δ*P*_CAM5_ for both OMEGA (*r* ^2^ = 0.97; Fig. 4a) and AMIP (*r* ^2^ = 0.94; Fig. 4e). Moreover, performance is consistent across all Δ*P*_CAM5_ values, with minimal conditional bias and only modest increases in the spread of the interquartile and 5–95% ranges moving towards large Δ*P*_CAM5_ values. The linear regression model dependent on *V*_m_ alone explains a substantial fraction of this variance (*r* ^2^ = 0.83), though not all of it (Fig. 4b, f). Inclusion of *f* and *r*_8_ as additional predictors largely eliminates this gap (Fig. 4c, g) for both OMEGA (*r* ^2^ = 0.95) and AMIP (*r* ^2^ = 0.95), consistent with the prevailing empirical model^6^. Note that the magnitudes of the MLR coefficients for *r*_8_ and *f* are not directly comparable across the simulations because of large differences in the covariance between the two parameters (Fig. 2). Finally, the theory-based MLR model dependent on *V*_m_ and the combined parameter $\frac{1}{2}f{r_8}$ performs equally well (Fig. 4d, h) for both OMEGA (*r* ^2^ = 0.94) and AMIP (*r* ^2^ = 0.95), providing strong evidence in favor of the theoretical prediction for the central pressure deficit presented above. MLR coefficients for (*V*_m_, $\frac{1}{2}f{r_8}$) are (1.79, 0.56) for OMEGA and (1.62, 0.61) for AMIP, indicating that OMEGA and AMIP exhibit quantitatively similar parametric dependencies despite large qualitative differences in their spatial distributions (Fig. 1) and distributions of relevant dynamical quantities (Fig. 2) as well as the wide range of additional environmental heterogeneity, including land, SST variations, and the full Earth-like array of modes of tropical and extratropical variability, found in AMIP that does not exist within OMEGA.

Similar results are obtained for the statistical models applied to observations. The linear regression model dependent on *V*_m_ alone explains most of the variance in Δ*P*_obs_ (*r* ^2^ = 87%; Fig. 4i). Inclusion of *f* and *r*_8_ as additional predictors improves the model (*r* ^2^ = 93%; Fig. 4j), with equivalent performance for the combined parameter $\frac{1}{2}f{r_8}$ (Fig. 4k) as was found in the simulations. MLR coefficients for (*V*_m_, $\frac{1}{2}f{r_8}$) are (1.57, 0.72), which are comparable to the values found in the two simulations.

Note that across both simulations and observations, the largest and smallest values of Δ*P*_CAM5_ are underpredicted by the statistical model, yet this does not occur for the GWB calculation in the model simulations. This finding suggests that the assumption of linearity in the regression may break down at these values; this issue is explored next. Moreover, in the low Δ*P*_CAM5_ limit, which generally corresponds to very weak storms, our methodology (e.g., storm tracker) may break down as well.

**Fig. 5:**
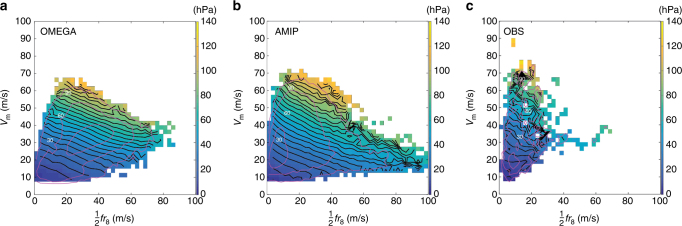
Joint dependence of Δ*P* on (*V*_m_, $\frac{1}{2}f{r_8}$) across simulations and observations. **a** OMEGA; binned MLR fit $\Delta {P_{{\rm{bin}}}} = - 25.9 + 1.82({V_{\rm{m}}}) + 0.46(\frac{1}{2}f{r_8})$ (${r^2} = 0.97,\;{N_{{\rm{bin}}}} = 528,\;{\epsilon _{{\rm{RMS}}}} = 4.9{\kern 1pt} {\rm{hPa}}$). **b** AMIP; binned MLR fit $\Delta {P_{{\rm{bin}}}} = - 25.2 + 1.77({V_{\rm{m}}}) + 0.57(\frac{1}{2}f{r_8})$ (${r^2} = 0.96,\;{N_{{\rm{bin}}}} = 641,\;{\epsilon _{{\rm{RMS}}}} = 5.5{\kern 1pt} {\rm{hPa}}$). **c** Observations; binned MLR fit $\Delta {P_{{\rm{bin}}}} = - 26.2 + 1.55({V_{\rm{m}}}) + 0.64\left( {\frac{1}{2}f{r_8}} \right)$ (${r^2} = 0.92,\;{N_{{\rm{bin}}}} = 281,\;{\epsilon _{{\rm{RMS}}}} = 7.8{\kern 1pt} {\rm{hPa}}$). Data are binned into 2.5 ms^−1^ increments increasing from zero for each parameter, with the median value of Δ*P* displayed in each bin (color). Black lines denote lines of constant Δ*P* with contour interval of 5 hPa. Purple lines denote bin count for *N* = [10, 100, 1000] for simulations and *N* = [3, 9, 27] for observations. All bins containing at least one data point are plotted

Indeed, while the linear models provide a simple and useful means of testing the theory, we may now discard assumptions of linearity and probe the full joint dependence of Δ*P* on *V*_m_ and $\frac{1}{2}f{r_8}$ in Fig. 5. This analysis is extended to include weak storms for which *V*_m_ < 15 ms^−1^. For both OMEGA (Fig. 5a) and AMIP (Fig. 5b), Δ*P*_CAM5_ monotonically increases primarily with *V*_m_ and secondarily with $\frac{1}{2}f{r_8}$. In accordance with the regression results of Fig. 4, this joint dependence is approximately linear in each direction over much of the phase space. Coefficients for the two-parameter MLR model with predictors (*V*_m_, $\frac{1}{2}f{r_8}$) fit to the binned data in Fig. 5 are (1.82, 0.46) for OMEGA (binned *r* ^2^ = 0.97) and (1.77, 0.57) for AMIP (binned *r* ^2^ = 0.96). The MLR fit for OMEGA with the data set restricted to $\left| \phi \right|$ < 40°, corresponding to the approximate latitude range found in AMIP and observations, yields coefficients for (*V*_m_, $\frac{1}{2}f{r_8}$) of (1.78, 0.57), which is similar to the values found for the full distribution and very close to the AMIP values. The results are similar to the values noted above for the unbinned data, though with a slightly stronger dependence on *V*_m_. Though linearity provides a good approximation, departure from linear dependence is evident in the increase in contour gradient magnitude moving from low to high values of Δ*P*_CAM5_, which will manifest as an underestimation of high Δ*P* by a linear model as noted above. Moreover, some curvature is evident towards the extremes of the phase space, though caution should be exercised specifically at the periphery of the phase space where sample sizes decrease towards zero. For large *V*_m_ and small $\frac{1}{2}f{r_8}$ in AMIP, the relative dependence on $\frac{1}{2}f{r_8}$ increases. This result may be due to super-gradient effects, whose magnitude can increase at high curvature^35,36^. More generally, this non-linearity may explain prior work identifying reduced performance in existing empirical wind-pressure relationships at high intensity^37^. For large $\frac{1}{2}f{r_8}$ and moderate *V*_m_ in OMEGA, the relative dependence on *V*_m_ increases, perhaps suggesting asymptotic behavior in the combined effect of size and rotation rate on Δ*P* at particularly large values. We note that curvature effects at the extremes in Fig. 5 will be poorly represented in a statistical model fit to the entire data set given that these regions of the phase space represent a small fraction of the total sample size.

Figure 5 further demonstrates that the joint dependence of Δ*P*_obs_ on *V*_m_ and $\frac{1}{2}f{r_8}$ in observations (Fig. 5c) is quantitatively similar to that found in OMEGA and AMIP. MLR coefficients for (*V*_m_, $\frac{1}{2}f{r_8}$) fit to the binned data are (1.55, 0.64) (binned *r* ^2^ = 0.92), a result comparable to that of AMIP. It is important to note that the observational database spans a much smaller subset of the phase space than any of the simulations. This contrast is associated with the combination of a wider simulated range in $\frac{1}{2}f{r_8}$ in the models and the restriction of the observational geographic domain primarily to the western North Atlantic basin, which limits the range^38^ of both *f* and *r*_8_ relative to AMIP. Nonetheless, the subset of the phase space spanned by the observations is nearly fully contained within that of the simulations, particularly AMIP. Indeed, for a more direct comparison of AMIP and observations, the MLR fit restricted to the phase space subset of valid observational bins within *V*_m_ ≤ 70 ms^−1^ and $\frac{1}{2}f{r_8} \le 30\,{\rm{m}}{{\rm{s}}^{ - 1}}$ yields coefficients for (*V*_m_, $\frac{1}{2}f{r_8}$) of (1.53, 0.73) for observations and (1.62, 0.79) for AMIP. Moreover, the solution for the functional dependence of Δ*P* on *V*_m_ and $\frac{1}{2}f{r_8}$ appears to vary smoothly when moving into the observational region of the phase space in both simulations.

Taken together, the convergence of results across the model hierarchy from reduced-complexity to real-world provide strong evidence in favor of a common underlying solution for the dependence of the central pressure deficit in nature. At a minimum, the results are mutually consistent and do not indicate the existence of a sudden regime change at the boundary between our experimental worlds and nature that might invalidate the application of these results to real-world storms.

## Discussion

Here we have derived, from gradient wind balance, a novel theoretical prediction that the central pressure deficit in a tropical cyclone depends principally on two velocity scales: the maximum azimuthal-mean azimuthal wind speed and half the product of the Coriolis parameter and a measure of outer storm size. The latter represents the planetary tangential velocity component of the planetary angular momentum available to the outer circulation of the storm. This prediction is then demonstrated to perform well across a hierarchy of models spanning global numerical simulation experiments and real-world observations. This hierarchy was designed to comprehensively test the prediction and to bridge the gaps from reduced-complexity models to real-world storms. Our experiments do not seek to accurately reproduce any particular historical climatology but rather to probe the fundamental nature of the tropical cyclone. Viewed as tropical cyclone factories, the simulations generate statistics that are much broader (parameter range) and deeper (sample size) than are accessible when limited strictly to the real world. Quantitatively similar results are obtained in a historical observational database, whose construction is similar to that employed in the prevailing empirical model for this relationship^6^.

These results likely explain why economic damages are better predicted by variations in the central pressure than by peak storm wind speed^26^, as the central pressure is essentially an integrated measure of the wind field that combines maximum wind speed and storm size; the latter is known to be a critical factor in damage potential, particularly due to storm surge^8,9,39,40^. Indeed, the central pressure is a single well-estimated quantity that is simpler than energy-based measures of the wind field^41^ that require accurate wind speed data over the entire storm. Moreover, these results may explain why climate models can reproduce the observed range of central pressure deficit despite cutting off the upper end of the distribution of peak wind speed, as lower resolution tends to produce weaker but larger storms^16,42–45^, whose competing effects on the central pressure deficit may largely offset. Finally, our results lend further support for the simple theoretical wind structure model exploited above, in particular the fast adjustment timescale of the structure of the wind field relative to that of outer storm size and intensity. This fast timescale underlies the prediction that the radial integral of gradient wind balance over the full wind field may be reduced simply to a dependence on maximum wind speed and a single measure of outer storm size as is borne out in our analysis.

There are a number of additional avenues that may warrant further research. First, though gradient wind balance appears to perform well in our analysis, gradient wind imbalance is known to occur in the vicinity of the eyewall and may induce secondary effects on the central pressure deficit not accounted for here. Second, resolution limitations prevent our model simulations from capturing particularly small, intense storms occasionally found in nature, and it is possible that the dependence on these parameters may yet differ moving towards the small size limit. Experiments in high-resolution simulations could provide additional evidence in this regime. Resolution limitations may also minimize more complex variability in the inner-core wind structure, such as eyewall replacement cycles and small-scale variability within the eye, that may further modulate the central pressure deficit. Finally, experiments testing the independent effects of varying surface enthalpy and momentum exchange coefficients on these dependencies may yield further insights, though our results do not appear to be strongly dependent on the details of their representation given that these coefficients are implicitly allowed to vary in CAM5.

For practical applications such as operations, additional work is required to develop an optimal predictive model for the central pressure deficit in nature; here we have focused on the basic underlying physics. Though the character of the functional dependence of Δ*P* is found to be similar across experiments and observations, the absolute values of that dependence may vary in more complex ways in nature as well as in alternative computer models. This variability reflects the uncertainty inherent in the messy details of boundary layer processes, represented crudely here by our wind rescaling factor for the gradient wind balance calculations; these processes can exert a strong and variable influence on how wind speeds vary with altitude relative to *z* = 10 m. Moreover, we do not address the specific operational need to predict the local (point) maximum wind speed, which must account for, e.g., the effects of azimuthal asymmetries in the wind field that can modulate peak local wind speed with minimal impact on both the azimuthal-mean wind speed and the central pressure deficit. Nonetheless, this same modeling framework may ultimately be useful for estimating the complete TC vortex, a WMO recommendation^46^, from the limited operationally available observations; this is a topic for future application development.

Overall, to our knowledge this is the first effort to explicitly test the fundamental physical relationship between the central pressure deficit of a tropical cyclone and its wind field. The apparent convergence of theory, reduced-complexity modeling experiments, Earth-like model simulations, and historical observations provides strong evidence for a fundamental, physically-intuitive underlying relationship among the two common measures of intensity (maximum wind speed, minimum central pressure), outer storm size, and latitude (Coriolis). The result has significant value for operational forecasting, risk assessment, and basic research and understanding of the tropical cyclone. For example, this framework may be applied to the evaluation of tropical cyclone climatologies and their intercomparison across climate models on the basis of the joint variability in these storm properties. Moreover, given independent measurements of maximum wind speed and minimum central pressure, these results may be used to infer storm size in the early-period historical record in the absence of modern remotely-sensed and in situ measurements.

More broadly, we highlight that this work seeks insight into the behavior of tropical cyclones on Earth in part via their analysis within alternative worlds. Indeed, OMEGA is a plausible yet imaginary world, perhaps analogous in certain respects to a planet such as Jupiter that is principally heated uniformly from within rather than non-uniformly by an external star. The combination of such reduced-complexity experiments with Earth-like simulations offer the best hope^30^ for improving the understanding and predictability of real storms in nature simultaneously, an objective that is otherwise difficult to achieve via the simulation of the real Earth and its myriad complexities alone.

## Methods

### Experimental design

We test the theoretical prediction for the dependence of Δ*P* across a hierarchy of models over a range of complexities that spans a reduced-complexity aquaplanet simulation experiment (OMEGA), an Earth-like simulation (AMIP), and real-world observations. The hierarchy is designed with two principal purposes: to provide a clean experimental testing ground in which parametric and phenomenological complexity is minimized (i.e., reduced-complexity) while retaining essential modes of variability; and to explicitly connect results from reduced-complexity experiments to those of a real-world setting. These simulation experiments are treated essentially as tropical cyclone factories containing thousands of snapshots of the phenomenon of interest. Our objective is not to accurately reproduce any particular historical climatology of tropical cyclones but rather to test hypotheses about the fundamental nature of the tropical cyclone in general.

### Simulation experiments

The first experiment, OMEGA, is a global radiative-convective equilibrium simulation with horizontally uniform sea surface temperature (29 °C) and solar insolation (340 Wm^−2^ diurnal-mean)^16^; this set-up is similar to previous global aquaplanet experiments^17,47^. The mean state is a world in which tropical cyclones are the dominant form of internal variability in the system. The spatial distribution of tropical cyclones is approximately zonally symmetric (Fig. 1) with storms that typically follow Earth-like poleward tracks due to the effect of beta drift^48–50^. Storms exist within a tightly controlled global climate state characterized by a homogeneous thermodynamic environment devoid of land, extratropical jet interaction, or other features that may inhibit storm formation or propagation. As a result, storm tracks are capable of extending all the way to the poles. Experimentally, then, OMEGA generates storms with a wide range of variability in *V*_m_, *r*_0_, and *f* within an otherwise homogeneous environment, which offers a clean setting in which to test our theory. The second experiment, AMIP, is an Earth-like historical simulation (i.e., following Atmospheric Model Intercomparison protocols^51^) over the period 1979–2012; this set-up was examined in previous work^15,52^. AMIP builds on OMEGA by adding full Earth-like environmental heterogeneity, including land masses and a jet stream, resulting in the confinement of storm tracks to specific ocean basins (Fig. 1) akin to the spatial distribution found on present-day Earth. A third experiment was also run that is identical to OMEGA except with the Coriolis parameter set to its value at *ϕ* = 10 °N everywhere on the planet, thereby imposing uniform dynamical forcing in the system; this experiment yields more complex results and is discussed in Supplementary Note 1 (Supplementary Figs 1, 2).

Our experimental laboratory is the Community Atmosphere Model, version 5 (CAM5). As the atmospheric component of the Community Earth System Model (CESM), CAM5 is typically used for conventional climate simulations. The spectral element dynamical core on a cubed-sphere grid^53,54^, used for all simulations, has demonstrated an ability to simulate tropical cyclones in a range of experimental setups^16,52,55,56^. All three simulations are run at a resolution of ne120, where “ne” denotes the number of spectral elements along each cube edge. This resolution yields a horizontal grid spacing of ~25 km that is nearly uniform around the globe owing to the cubed-sphere grid of the dynamical core. The same physical parameterization suite^57^ is employed across all experiments, with the exception of various simplifications required for the reduced complexity of OMEGA^58^. CAM5 employs a hybrid-sigma model vertical coordinate whose lowest model levels conveniently are nearly equivalent to levels of constant altitude when surface elevation is constant. OMEGA was also run at a lower horizontal resolution of 100 km and yields similar conclusions (Supplementary Figs 3, 4). OMEGA was run for 2 years, while AMIP spans the period 1979–2015. To allow for model equilibration, the first six simulation months are discarded for OMEGA and the first year (1979) is discarded for AMIP.

For each simulation, tropical cyclones are first identified using a tracker that also extracts the surface central pressure at the storm center. For OMEGA, the storm center is determined on the quasi-uniform native cubed-sphere grid using an early release version of the open-source TempestExtremes^59^. Given the simplicity of the simulations, the algorithm searches for a surface pressure minimum that includes a 4-hPa closed contour within five great circle degrees of the location of the minimum at 6-hour increments. The storm locations are then stitched together by searching for candidates at the next 6-hourly time step that are within five great circle degrees. A track is defined as having at least four storm points, with gaps between consecutive track points not exceeding 24 hours. For AMIP, due to its greater phenomenological complexity, the storm center is tracked at three-hour increments with an alternate algorithm^60^ commonly used for Earth-like simulations. This algorithm associates storm centers on a latitude-longitude grid (via bilinear interpolation from the native grid) by matching a local minimum in surface pressure with a maximum in relative vorticity at 850 hPa and a warm-core between 500 and 300 hPa. These storm centers are then stitched together by looking to the next time step for another center that is within 400 km. A track is defined to have a lowermost model level wind speed great than 17 m s^−1^ for a minimum of three days. All analysis throughout is performed on the native grid, which requires simple relocation of the storm center to the native grid for AMIP (due to the tracking being performed on a latitude-longitude grid).

For each identified center at each time step, azimuthal-mean radial profiles of azimuthal wind and pressure are calculated from data on the native grid at the lowest model level out to 5000 km radius. Radial profiles of density were also calculated for OMEGA, but the requisite data was not available for AMIP. Data are binned into Δ*r* = 25 km bins in increments of $\frac{1}{4}\Delta r$. For the AMIP simulations, data are first blocked out at all gridpoints where the CAM5 land fraction variable is >0.1 and the surface geopotential height is outside the range *z* ∈ [−10, 10] *m* in order to minimize biases induced by land or elevated terrain as well as to ensure that the lowest hybrid-sigma model level corresponds closely to a surface of constant altitude.

Given that the relationship between theory and prediction is mediated by gradient wind balance, we first test the extent to which gradient wind balance may be directly applied to the full radial profile of azimuthal-mean azimuthal wind in order to predict the central pressure deficit of the storms. The radial profile of the azimuthal-mean azimuthal wind is first multiplied by a constant factor for each simulation (values given in the main text); this factor is interpreted here as a simple accounting for the reduction in wind speed due to friction within the boundary layer (i.e., the reciprocal of the gradient-to-surface wind reduction factor^61^). This approach avoids the many complexities of defining the top of the boundary layer^62^ and aligns with the overarching objective in operations and research of relating the near-surface maximum wind speed to the central surface pressure and vice versa. This wind profile is interpolated to a 1-km resolution radial grid using a Piecewise Cubic Hermite Interpolating Polynomial, which is similar to a cubic spline but prevents overshoot between neighboring data points. Given this wind profile, the gradient wind equation (Eq. 1) is integrated radially outward from the center to the first radius of vanishing wind, *r*_0_, in 2-km increments using a centered-difference scheme to yield a prediction for the storm central pressure deficit Δ*P*_GWB_. Air density is set constant at *ρ* = 1.155 kgm^−3^; inclusion of the full radial profile of density in the GWB integration for OMEGA does not significantly change the result (Supplementary Fig. 5). This prediction is compared against the true central pressure deficit, Δ*P*_CAM5_, with the environmental pressure defined as *P*_0_ = *P*(*r* = *r*_0_) from the radial profile of pressure. For a small fraction of cases, the azimuthal wind gets very close to zero but does not cross it; in such cases the largest valid integer wind radius (e.g., radius of 1 ms^−1^) of the raw CAM5 wind profile is used instead. The wind profile at small wind speeds in the far outer circulation tends to exhibit significantly more variability, which may introduce additional noise in our analysis that might be avoided by applying an outer wind model instead^31^. However, Δ*P* itself is relatively insensitive to these very weak winds at very large radii due to the nature of Eq. (1), and thus we elect to use the wind profile alone for the sake of simplicity. Examples of the methodology applied to one storm from each of OMEGA and AMIP are provided in Fig. 2.

Next, we test the extent to which the results of the gradient wind balance prediction can be replicated by a simple statistical multiple linear regression model that employs the parameters identified above by theory: *V*_m_ and $\frac{1}{2}f{r_0}$. In contrast to the gradient wind balance calculation, large variability at small wind speeds in the far outer circulation will have an outsized effect on the statistical model prediction, and thus in lieu of *r*_0_ we employ the inner-most radius of 8 ms^−1^, *r*_8_, as our measure of outer storm size (Fig. 3). This choice has the added benefit that *r*_8_ typically lies well above the noise of the background environmental flow; typically lies sufficiently far outside the radius of maximum wind (even for relatively weak storms) so as to lie beyond the turbulently convecting inner core; and may potentially be observed in situ or through remote sensing or calculated from reanalysis data and thus be accessible in an operational setting. Moreover, *r*_8_ performs best in comparison of the outer wind field between reanalysis and observations^63^. Three versions of the statistical model are tested, each taking different input parameters: *V*_m_ only; *V*_m_, *f*, and *r*_8_; and *V*_m_ and $\frac{1}{2}f{r_8}$. The first model provides a pure wind-pressure model as a baseline; the second model adds information about *f* and *r*_8_ separately and is analogous to the prevailing empirical model^6^; the third model tests the theoretical prediction that the effects of *f* and *r*_8_ manifest themselves as a single joint parameter $\frac{1}{2}f{r_8}$.

The above tests are performed using subsets of storm snapshots filtered to exclude cases with *V*_m_ < 15 ms^−1^ to avoid very weak storms, as well as rare instances where *r*_m_ < 10 km or *r*_m_ > 500 km; the latter may occur briefly in OMEGA if a weak storm is very close to a strong storm. For AMIP simulations, cases in which the center is less than 100 km from land are also excluded in order to minimize land effects on the wind profile. Furthermore, to avoid cases with poor azimuthal data coverage, an asymmetry parameter^38^, whose values range from zero (perfect azimuthal symmetry) to one (single point), cannot exceed 0.5 at *r*_8_; this filters out ~1% of cases and has a negligible effect on the results. Filters are calculated from the raw CAM5 radial profiles of the azimuthal wind.

### Observations

Finally, atop the model hierarchy is the our best estimate of the real Earth itself. We construct an aircraft-based observational data set for 2004–2015 from the Atlantic and East Pacific basins (Fig. 1) using a similar methodology to the prevailing empirical model^6^. Tropical cyclone Best Track intensity and positions are interpolated to the time of aircraft-based estimates of central pressure. The interpolated intensity is slightly reduced^64^ to account for the effect of storm motion by removing the asymmetry, *a*, owing to storm motion, *c*, given by *a* = 1.173*c* ^0.63^ ms^−1^. Both Best Track and aircraft reconnaissance central pressure fixes come from the databases of the Automated Tropical Cyclone Forecast system (ATCF^65^) available from the NHC. To estimate *r*_8_, Global Forecast System (GFS) operational analyses are utilized to calculate the radial profile of 850 hPa winds using adjacent analysis times and linearly interpolating to a single radial profile. 850-hPa winds are reduced to a marine exposure using a factor of 0.80. The value of *r*_8_ is defined as the inner-most wind radius beyond the radius of maximum wind, and the algorithm saturates at 1500 km; a small subset of values associated with Sandy (2012) attain this upper bound and thus may be slightly underestimated. The environmental pressure is estimated by the azimuthal-mean pressure at 900 km (*r*_8_ < 600 km), 1200 km (*r*_8_ ∈ [600, 900) km), 1500 km (*r*_8_ ∈ [900, 1200) km), 1800 km (*r*_8_ ∈ [1200, 1500) km), and 2100 km (*r*_8_ ≥ 1500 km). Finally, the central pressure deficit, Δ*P*_obs_, is calculated by subtracting the central pressure from the environmental pressure. As with AMIP, only cases in which the center is at least 100 km from land are included. Distributions of key quantities for the observational database are provided in Fig. 2.

### Code availability

All code used to perform the analyses in this work are available upon request from the corresponding author.

### Data availability

The data sets analyzed in this study are available from the corresponding author on reasonable request. All model output is accessible via the National Center for Atmospheric Research (NCAR) Yellowstone supercomputer.

## Electronic supplementary material

Supplementary Information

Peer Review File
