# Supplementary material for: Physical understanding of the tropical cyclone wind-pressure relationship
Source: Nat Commun. 2017 Nov 8;8:1360. doi: 10.1038/s41467-017-01546-9 (PMC5678138; doi:10.1038/s41467-017-01546-9)
Supplement: Supplementary file 1 — Supplementary Information [file 41467_2017_1546_MOESM1_ESM.pdf]

Supplementary Note 1

A third simulation experiment, FCNST, was run that is identical to the OMEGA experiment (see main manuscript for details), except with the Coriolis parameter set to its value at $\phi = 10\ °N$ everywhere on the planet. This experiment was analyzed in previous work[1] and was found to closely resemble that of f-plane rotating RCE simulations superimposed onto a spherical grid. FCNST produces meandering, long-lived storms whose spatial distribution is horizontally symmetric. Storms exhibit a wide range of variability in V_m and r_0 at fixed f within an otherwise homogeneous environment. Though FCNST is designed to be the simplest of the simulations, it yields a *more* complex result relative to that found for the rest of the model hierarchy as discussed below.

Supplementary Figure 1 compares the model prediction of ΔP against their true values for the direct gradient wind balance calculation and the statistical models for FCNST, analogous to Figure 4 of the main manuscript. For the gradient wind calculations, the azimuthal wind profile has been multiplied by the constant factor of 1.44, which is significantly larger than that found in the OMEGA and AMIP. The GWB calculation performs less well ($r^2 = 0.76$) than was found for the other simulations. In contrast, the statistical model performance is similar to that of the other simulations ($r^2 = 0.92$), with MLR coefficients for (V_m, $\frac{1}{2}fr_8$) of (2.19, 1.21); recall that the latter coefficient depends only on storm size, as f is constant. Supplementary Figure 2 displays the joint dependence of ΔP on V_m and $\frac{1}{2}fr_8$ for FCNST, analogous to Figure 5 of the main manuscript, with MLR coefficients for (V_m, $\frac{1}{2}fr_8$) fit to the binned data are (1.88, 1.16). This indicates a stronger dependence on $\frac{1}{2}fr_8$ than in the other simulations or observations.

This more complex behavior is not currently understood. Importantly, the FCNST simulation differs qualitatively from those simulations closer to the real world in one critical manner: storms meander very slowly over their very long (perhaps infinite) lifetimes, as there are no preferred directions of storm motion nor genesis/lysis regions such as is found in the other experiments and in nature. Additionally, the characteristic storm size is larger owing to the relatively low dynamical latitude employed for the constant value of f in this long-run equilibrium simulation[1]. We speculate that additional dynamical processes associated with the combination of long time-scales, slow storm motion, and/or larger sizes may alter the structure of the boundary layer in a unique manner that are negligible for storms exhibiting well-defined life-cycles. If such processes enhance the wind speed reduction between gradient level and the surface and this effect increases with storm size, then the result would be an enhanced dependence of the central pressure deficit on storm size as has been found here. Additional experimentation and analysis to understand this case is left for future work.

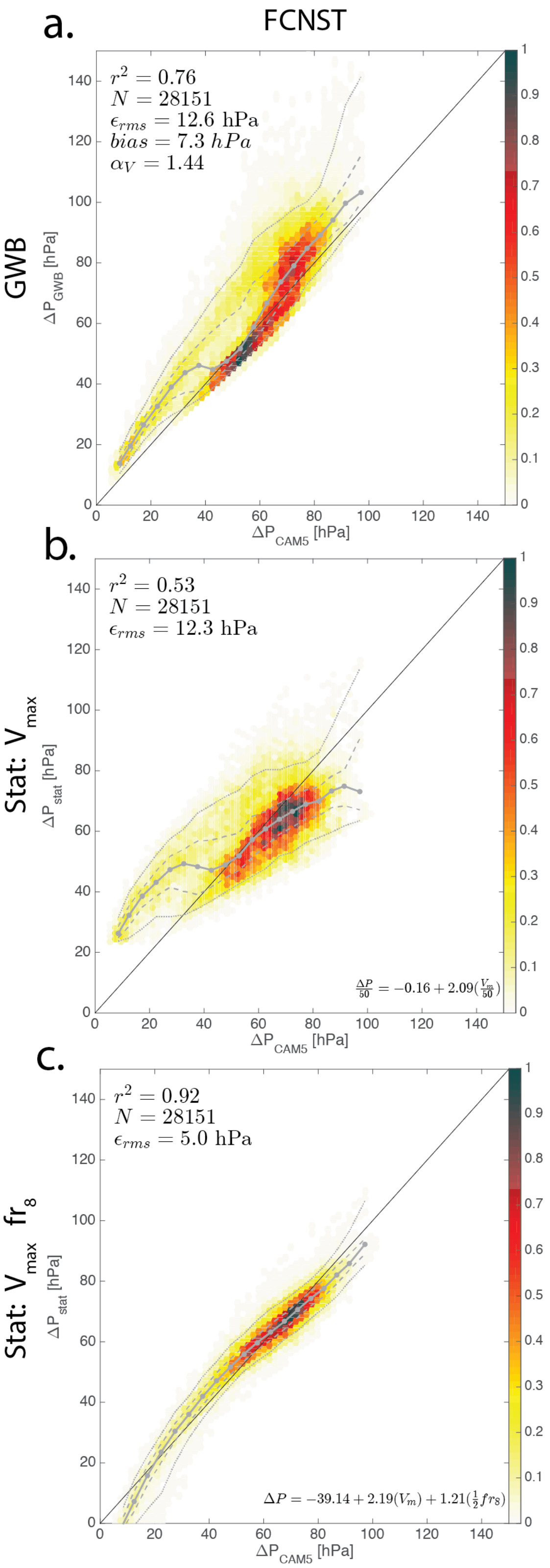

Supplementary Figure 1. Predicted vs. actual ΔP [hPa] for FCNST simulation. a. Direct gradient wind balance model. b. MLR model with predictor V_m. c. MLR model with two predictors (V_m, $\frac{1}{2}fr_8$). Color denotes relative frequency within hexagonal bin. Black line denotes one-to-one correspondence. Gray lines denote median (solid), interquartile range (dashed), and 5-95% range (dotted) of predicted ΔP within 5 hPa bins of ΔP_{CAM5} starting from zero, plotted at bin-median value of each (dot) for bins with at least five data points. N denotes sample size. For GWB, mean prediction bias defined as $\overline{\Delta P}_{GWB} - \overline{\Delta P}_{CAM5}$, and α_V denotes wind speed rescaling factor.

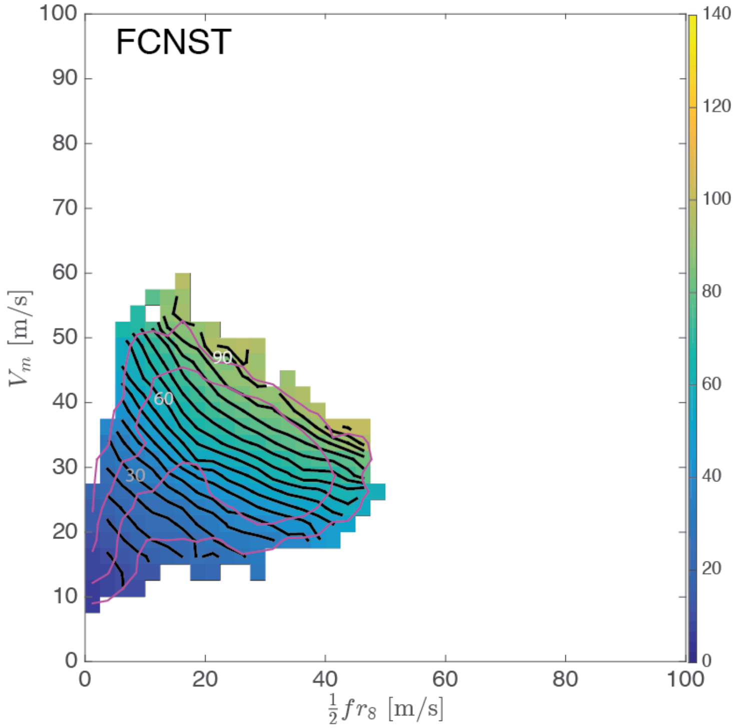

Supplementary Figure 2. Joint dependence of ΔP on (V_m, $\frac{1}{2}fr_8$) for FCNST simulation. Binned MLR fit ΔP_{bin} = -30.1 + 1.88(V_m) + 1.16($\frac{1}{2}fr_8$) ($r^2 = 0.96, N_{bin} = 243, \epsilon_{RMS} = 5.3\ hPa$). Data are binned into 2.5 ms^{-1} increments increasing from zero for each parameter, with the median value of ΔP displayed in each bin (color). Black lines denote lines of constant ΔP with contour interval of 5 hPa. Purple lines denote bin count for N=[10, 100, 1000]. All bins containing at least one data point are plotted.

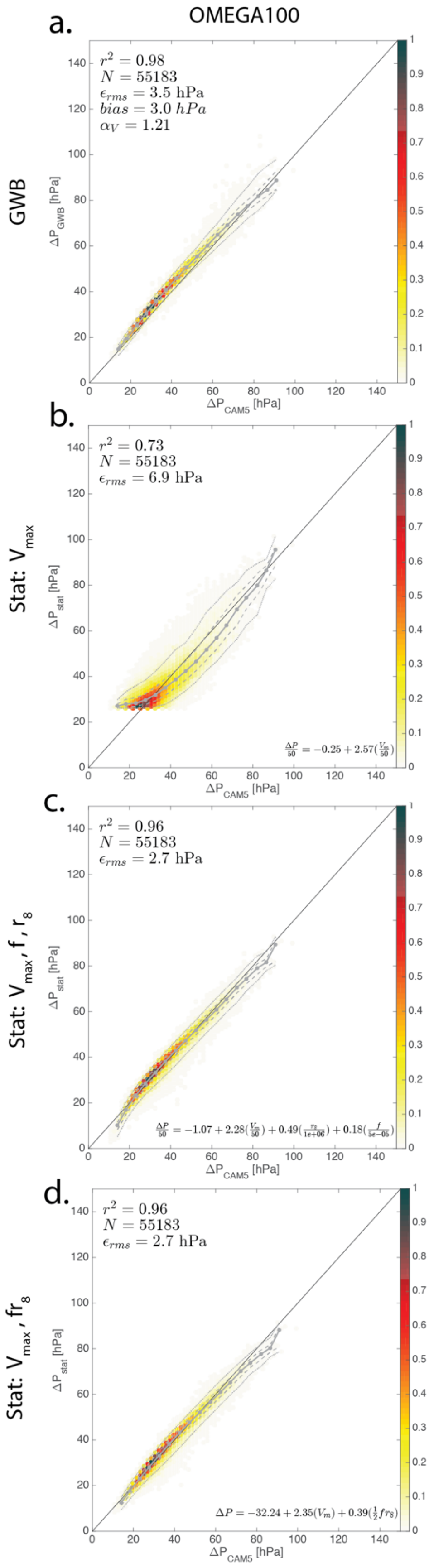

Supplementary Figure 3. Predicted vs. actual ΔP [hPa] for OMEGA100 simulation. a. Direct gradient wind balance model. b. MLR model with predictor V_m. c. MLR model with three predictors (V_m, f, r_8). d. MLR model with two predictors (V_m, $\frac{1}{2} f r_8$). Color denotes relative frequency within hexagonal bin. Black line denotes one-to-one correspondence. Gray lines denote median (solid), interquartile range (dashed), and 5-95% range (dotted) of predicted ΔP within 5 hPa bins of ΔP_{CAM5} starting from zero, plotted at bin-median value of each (dot) for bins with at least five data points. N denotes sample size. For GWB, mean prediction bias defined as $\overline{\Delta P}_{GWB} - \overline{\Delta P}_{CAM5}$, and α_V denotes wind speed rescaling factor. OMEGA100 simulation is analogous to OMEGA but at a lower horizontal resolution of $\Delta x \approx 100\ km$.

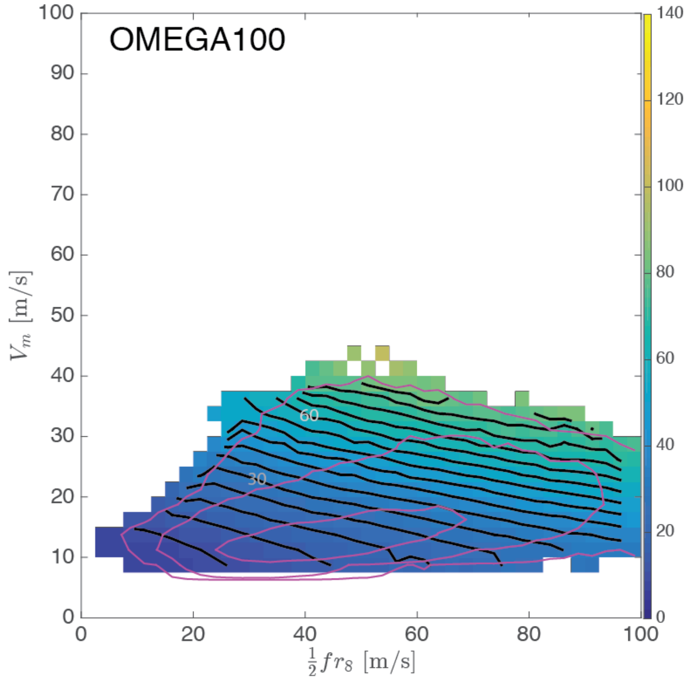

Supplementary Figure 4. Joint dependence of ΔP on (V_m, $\frac{1}{2}fr_8$) for OMEGA100 simulation. Binned MLR fit ΔP_{bin} = -26.1 + 2.13(V_m) + 0.39($\frac{1}{2}fr_8$) ($r^2 = 0.98, N_{bin} = 406$, $\epsilon_{RMS} = 3.4\ hPa$). Data are binned into 2.5 ms^{-1} increments increasing from zero for each parameter, with the median value of ΔP displayed in each bin (color). Black lines denote lines of constant ΔP with contour interval of 5 hPa. Purple lines denote bin count for N=[10, 100, 1000]. All bins containing at least one data point are plotted.

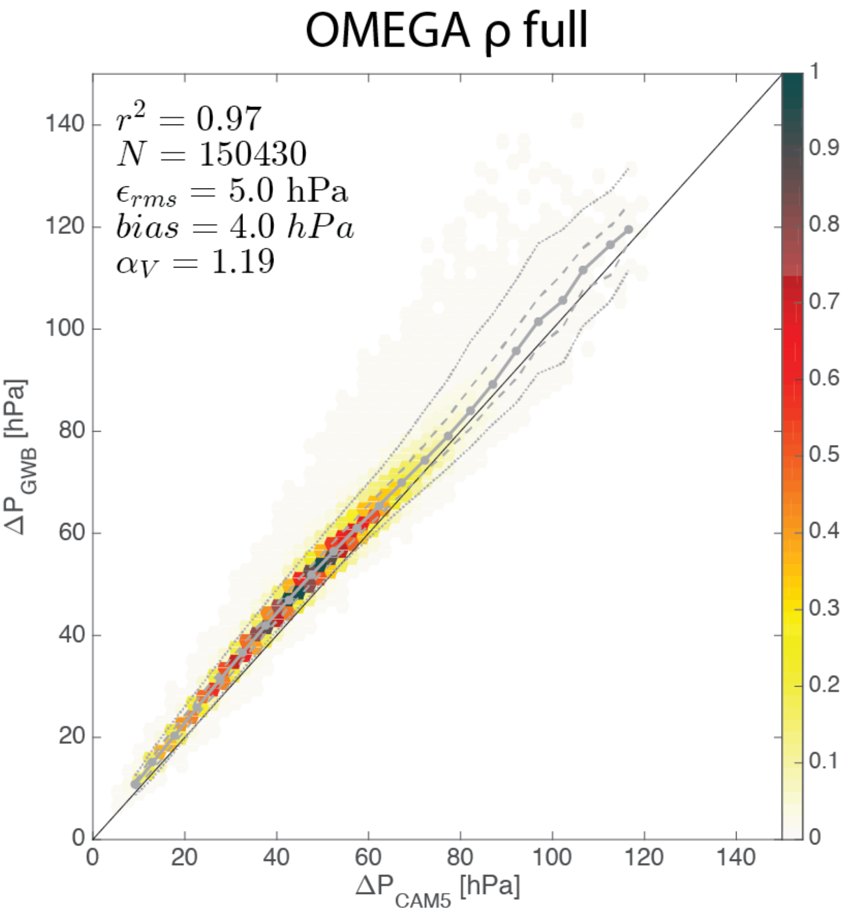

Supplementary Figure 5. Predicted vs. actual ΔP [hPa] for OMEGA simulation for the direct gradient wind balance model calculated using the full radial profile of density. Color denotes relative frequency within hexagonal bin. Black line denotes one-to-one correspondence. Gray lines denote median (solid), interquartile range (dashed), and 5-95% range (dotted) of predicted ΔP within 5 hPa bins of ΔP_{CAM5} starting from zero, plotted at bin-median value of each (dot) for bins with at least five data points. N denotes sample size. For GWB, mean prediction bias defined as $\overline{\Delta P}_{GWB} - \overline{\Delta P}_{CAM5}$, and α_V denotes wind speed rescaling factor.

Supplementary Reference

1. Reed, K. A., and D. R. Chavas, 2015: Uniformly rotating global radiative-convective equilibrium in the Community Atmosphere Model, version 5. Journal of Advances in Modeling Earth Systems, 7, 1938–1955, doi:10.1002/2015MS000519.
