# Supplementary material for: Physical understanding of the tropical cyclone wind-pressure relationship
Source: Nat Commun. 2017 Nov 8;8:1360. doi: 10.1038/s41467-017-01546-9 (PMC5678138; doi:10.1038/s41467-017-01546-9)
Supplement: Supplementary file 2 — Peer Review File [file 41467_2017_1546_MOESM2_ESM.pdf]

Editorial Note: this manuscript has been previously reviewed at another journal that is not operating a transparent peer review scheme. This document only contains reviewer comments and rebuttal letters for versions considered at Nature Communications.

REVIEWERS' COMMENTS:

Reviewer #1 (Remarks to Author):

This is my second review of this manuscript. On the first round of reviews, I had already given very positive comments about the manuscript, with only a few suggestions for the revisions. The authors have significantly improved the manuscripts taking into account both reviewers' suggestions. I'm very happy with this version of the manuscript and recommend the publication of the manuscript as it is. This is an excellent manuscript with important applications that should be published in a top journal.
